# A 77 Amino Acid Region in the N-Terminal Half of the HSV-1 E3 Ubiquitin Ligase ICP0 Contributes to Counteracting an Established Type 1 Interferon Response

**DOI:** 10.1128/spectrum.00593-22

**Published:** 2022-06-22

**Authors:** Mirna Perusina Lanfranca, Jessica M. van Loben Sels, Cindy Y. Ly, Tristan R. Grams, Adit Dhummakupt, David C. Bloom, David J. Davido

**Affiliations:** a Department of Molecular Biosciences, University of Kansas, Lawrence, Kansas, USA; b Department of Molecular Genetics and Microbiology, University of Florida College of Medicine, Gainesville, Florida, USA; Barnard College, Columbia University

**Keywords:** HSV-1, ICP0, interferon-beta, viral gene expression, E3 ubiquitin ligase, herpes simplex virus, innate immunity, interferons

## Abstract

Herpes simplex virus 1 (HSV-1) is a human pathogen capable of establishing lifelong latent infections that can reactivate under stress conditions. A viral immediate early protein that plays important roles in the HSV-1 lytic and latent infections is the viral E3 ubiquitin ligase, ICP0. ICP0 transactivates all temporal classes of HSV-1 genes and facilitates viral gene expression. ICP0 also impairs the antiviral effects of interferon (IFN)-β, a component of host innate defenses known to limit viral replication. To begin to understand how ICP0 allows HSV-1 to disarm the IFN-β response, we performed genetic analyses using a series of ICP0 truncation mutants in the absence and presence of IFN-β in cell culture. We observed that IFN-β pretreatment of cells significantly impaired the replication of the ICP0 truncation mutants, *n*212 and *n*312, which code for the first 211 and 311 amino acids of ICP0, respectively; this effect of IFN-β correlated with decreased HSV-1 early and late gene expression. This increased sensitivity to IFN-β was not as apparent with the ICP0 mutant, *n*389. Our mapping studies indicate that loss of 77 amino acids from residues 312 to 388 in the N-terminal half of ICP0 resulted in a virus that was significantly more sensitive to cells pre-exposed to IFN-β. This 77 amino acid region contains a phospho-SUMO-interacting motif or -SIM, which we propose participates in ICP0’s ability to counteract the antiviral response established by IFN-β.

**IMPORTANCE** Interferons (IFNs) are secreted cellular factors that are induced by viral infection and limit replication. HSV-1 is largely refractory to the antiviral effects of type 1 IFNs, which are synthesized shortly after viral infection, in part through the activities of the viral regulatory protein, ICP0. To understand how ICP0 impedes the antiviral effects of type 1 IFNs, we used a series of HSV-1 ICP0 mutants and examined their viral replication and gene expression levels in cells stimulated with IFN-β (a type 1 IFN). Our mapping data identifies a discrete 77 amino acid region in the N-terminal half of ICP0 that facilitates HSV-1 resistance to IFN-β. This region of ICP0 is modified by phosphorylation and binds to the posttranslational modification SUMO, suggesting that HSV, and potentially other viruses, may counteract type 1 IFN signaling by altering SUMO and/or SUMO modified cellular proteins.

## INTRODUCTION

Herpes simplex type 1 (HSV-1) is a ubiquitous pathogen that infects between 60% to 80% of the human population worldwide. Herpetic infections range from fever blisters or vesicular eruptions around the mouth, called cold sores, genital infections, to blindness and encephalitis (1–3). HSV-1 viral infection poses a severe risk for immunocompromised individuals (4). In this population HSV-1 has high mortality and morbidity (1), as a result of a higher incidence for lethal viral encephalitis (5).

A hallmark of HSV-1 infections is the ability of the virus to infect and establish a lifelong latent infection in the sensory neurons of the trigeminal ganglia (6). Latency is characterized by the absence of infectious virus while the viral genome persists in the neurons. Under conditions of stress HSV-1 can reactivate from latency leading to the production of progeny virus (1). During productive infection, HSV-1 has a temporal gene expression cascade categorized as immediate early (IE), early (E), and late (L) expression. Among the IE proteins expressed, infected cell protein 0 (ICP0) is required for efficient lytic viral replication by stimulating all classes of viral genes (7) and regulating the switch between the lytic and latent stages of infection.

ICP0 possesses E3 ubiquitin ligase activity through its RING-finger motif, which is associated with its ability to stimulate HSV-1 gene expression (8–10). As an E3 ubiquitin ligase, ICP0 attaches and polymerizes ubiquitin chains to proteins, typically marking them for degradation by the proteasome (11–14). ICP0 is capable of inactivating an intrinsic defense complex, nuclear domain 10 (ND10), through the degradation of several ND10 constituents, such as promyelocytic leukemia protein (PML), Sp100, and their SUMOylated isoforms (reviewed in Ref. 9). SUMOylated ND10 constituents like PML appear to be degraded, in part, through involvement of ICP0's SUMO-interacting motifs (SIMs) or SIM-like sequences (SLSs) (15, 16). These regions allow ICP0 to bind to SUMO isoforms and SUMO-conjugated protein(s), leading to their ubiquitination and subsequent degradation. ICP0 is also highly phosphorylated, and known sites of ICP0 phosphorylation are associated with its ability to dissociate ND constituents, conjugate ubiquitin, and transactivate viral genes in cell culture (17–19). ICP0 phosphorylation sites mutants are impaired for HSV-1 gene expression or lytic replication in cell culture (17, 19) and acute replication and reactivation in a mouse model of HSV-1 infection (6). Collectively, these studies indicate that ubiquitination, SUMOylation, and phosphorylation are important determinants that regulate or are regulated by ICP0.

Relevant to this current study, ICP0 confers to HSV its resistance to antiviral factors known as type 1 interferons (IFNs), members of innate defenses. IFNs (20, 21) are a family of cytokines capable of interfering with viral infections in the host (22) that are induced by viruses (23). There are three classes of IFNs in humans, from which two members of the type 1 family (i.e., α, β) are synthesized shortly after viral attachment or penetration and trigger an immune response (24–26). These IFNs are secreted and activate the JAK-STAT signal transduction cascade, stimulating the expression of hundreds of ISGs (interferon stimulated genes); a subset of proteins encoded by these genes are known to affect viral mRNA stability, processing, and translation of viral mRNAs (27). During lytic and quiescent infections, ICP0 has been shown to decrease histone binding and repressive histone modifications and to increase histone acetylation on HSV-1 promoters, alterations associated with enhanced viral gene expression (28, 29). At least two studies have indicated that the ND10-associated protein PML expression is stimulated by type 1 IFNs (i.e., an ISG), directly linking one aspect of intrinsic to innate defenses (20, 30, 31). Additionally, IFNs can also activate the adaptive immune response of the host, providing yet another mechanism by which type 1 IFNs limit viral replication.

Many viruses have developed counter-measures against IFNs, allowing for viral persistence and transmission (32). In the case of HSV-1, ICP0 plays an important role in counter-defenses. It has been shown that ICP0 expression is able to interrupt the ISG upregulation typically triggered by viral infection (33). ICP0’s role in modulating a preexisting type 1 IFN response was established in several studies showing that different ICP0 null mutants are highly sensitive to the effects of IFN-α or -β in cell culture, as well as in a mouse model of HSV-1 infection (34–40). This deficiency can be complemented in cell culture studies by exogenous expression of wild type (WT) ICP0 (33).

The overall mechanisms by which ICP0 impairs the established IFN response of the host are largely unknown. We performed mutational analyses on *ICP0* to begin to identify such mechanisms at the molecular level and to define one or more domains required for this activity. Using replication and gene expression assays in cell culture, we identified that the first 388 N-terminal amino acids of ICP0 are involved in the impairment of the IFN-β response, with residues from 312 to 388 (a 77 amino acid region) within the N-terminal portion of ICP0 required for this activity.

## RESULTS

### A region between amino acids 212 and 427 within the N-terminus of ICP0 is involved in the impairment of the interferon response.

**FIG 1:**
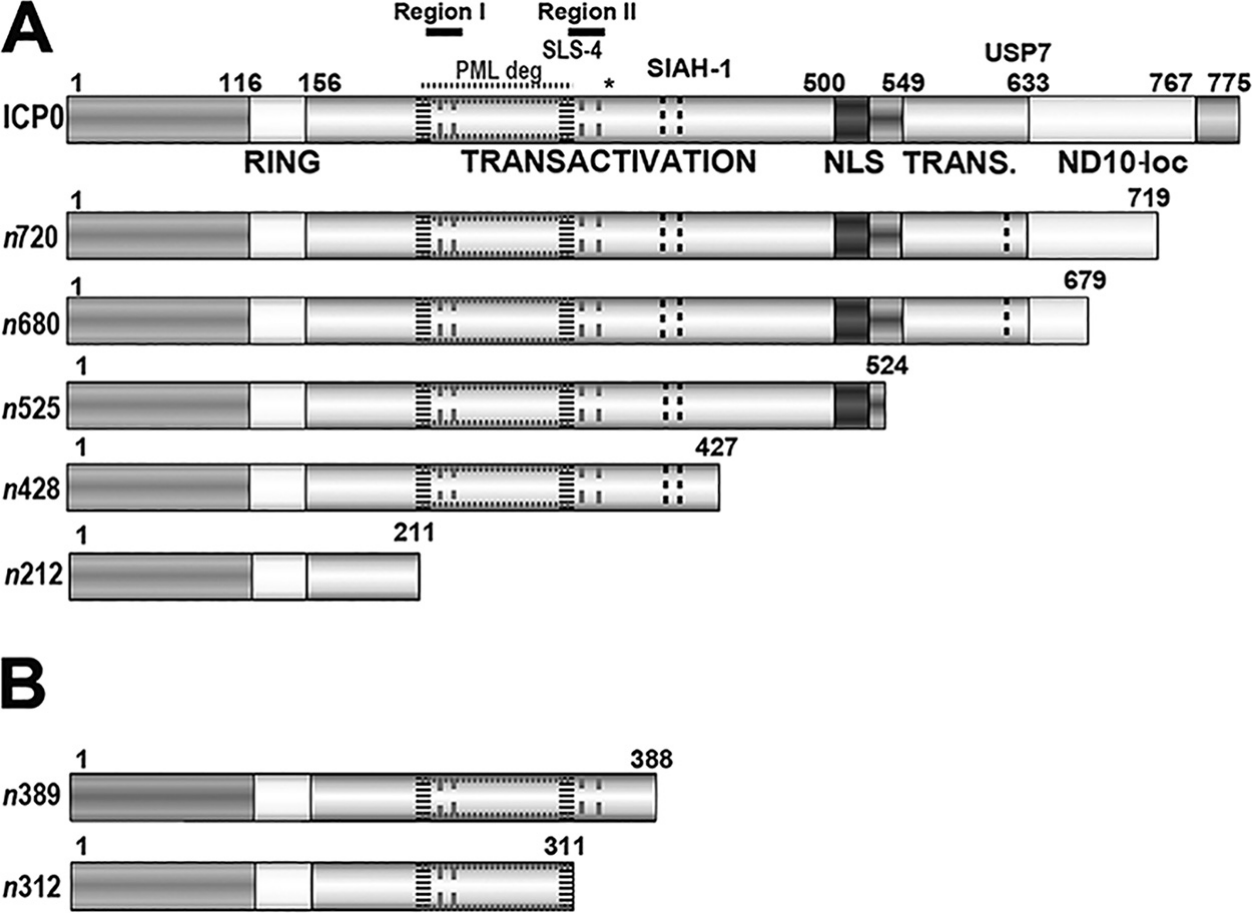
Functional domains of ICP0 and ICP0 truncation mutants. (A) Schematic of WT ICP0, expressed from WT HSV-1, and mutant forms of ICP0 expressed from the viruses *n*720, *n*680, *n*525, *n*428 and *n*212 are shown above; each ICP0 mutant contains a nonsense linker insertion within the ICP0 gene (85). Selected domains and their amino acid residue boundaries are shown: a RING-finger motif, a central transactivation domain, a nuclear localization signal (NLS), a C-terminal transactivation domain (TRANS), which includes an ND10 localization sequence (ND10-loc). Other domains include phosphorylated region I (224-232), PML degradation domain (212-312), SLS-4 (asterisk, 362-364), phosphorylated region II (365-371), a SIAH-1 binding site (400-410), and a USP7 binding site (618-638). (B) Schematic of additional mutant forms of ICP0 generated as previously described (66).

As previously mentioned, ICP0 is crucial for the ability of HSV-1 to overcome an established type 1 IFN response. Initially using ICP0 C-terminal truncation mutants generated by the Schaffer Laboratory (Fig. 1A), WT HSV-1, and an ICP0 null mutant (7134), we performed plaque reduction assays in three cell types in the absence and presence of IFN-β. Of the cell types examined, we used Vero (African green monkey kidney) cells, which are responsive to IFN treatment but unable to synthesize it (41). Additionally, we chose a primary cell strain, human embryonic lung (HEL) 299 cells, for these experiments as HEL cells are capable of responding to and synthesizing type 1 IFNs resulting from viral infection (42, 43). Lastly, a Vero complementing cell line (L7 cells), which expresses ICP0 upon infection, was included in this study to confirm that ICP0 was responsible for the plaquing phenotypes we observed with our ICP0 mutants in Vero cells.

**FIG 2:**
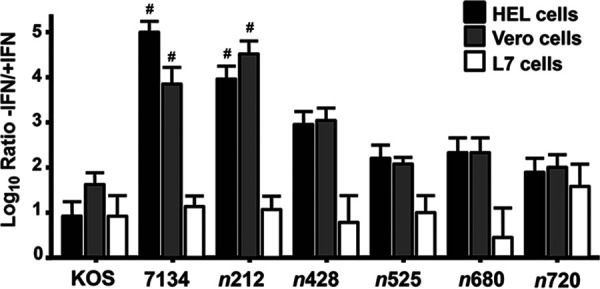
WT HSV-1 and ICP0 null and truncation mutants in plaque reduction assays in the presence and absence of IFN-β. HEL, Vero, and L7 cells were plated in 24-well plate and either mock-treated or pretreated with 1000 U/mL of human IFN-β. After 15 h of IFN-treatment, cells were infected with 10-fold serial dilutions of the WT (KOS), ICP0 null (7134), and C-terminal truncation mutant viruses. Three days pi cells were fixed and stained by immunohistochemistry with a polyclonal anti-HSV-1 antibody. Ratios were determined as PFU/mL in −/+IFN for KOS, 7134, and C-terminal truncation mutants. Pound signs (#) denote samples with no visible plaques at lowest dilution +IFN; these samples were given the value of 1 to estimate −/+ IFN ratios. Error bars represents standard errors of the means (SEMs). Experiments were repeated between 6- to 14-times for given cell lines and viruses.

When we evaluated the plaque reduction assay results performed in the presence of IFN-β and in all three cell lines, we noticed that there was a reduction in plaque size for all viruses tested compared to the ones performed in the absence of IFN-β (M. Perusina Lanfranca and D. J. Davido, unpublished data). These results were similar to a published report indicating that IFN-β impacts HSV-1’s ability to spread from cell-to-cell, whether the virus is WT HSV-1 or an ICP0 null mutant (44). We then compared the plaquing efficiencies of each virus on the 3 cell lines by taking the ratio of viral titers in the absence of IFN-β versus in the presence of IFN-β. Consequently, the higher the ratio the greater difficulty a virus has plaquing in IFN-β-treated cells. For the WT strain KOS, the average ratio in HEL cells was 6.5-fold. Fold differences for *n*428 to *n*720 in HEL cells ranged from 50- to 400-fold, whereas it was difficult to visualize plaques for *n*212 and the ICP0 null mutant, 7134, upon IFN-β pretreatment. These trends were very similar in Vero cells (Fig. 2). Because individual plaques could not be detected with 7134 (ICP0-null mutant) and *n*212 upon the addition of IFN-β, the lowest dilution of a virus tested was given the value of 1 to estimate reductions in the plaquing efficiency of these viruses (indicated by pound signs). Thus, decreases in the ratios of *n*212 and 7134 plaquing were estimated to be ≥3 × 10^3^ fold. Our results in Fig. 2 indicate that there was a clear-cut-off in the plating efficiency between *n*212 and *n*428 that was dependent on IFN-β, given that individual plaques were not visible in *n*212 IFN-β-treated cells. We also examined whether the ND10-associated protein, PML, played a role in this IFN restriction. It has been shown that PML is an ISG (45–47). To assess PML’s role, we performed plaque reductions using control and PML-depleted cells with the ICP0 mutants described in Fig. 2. Our data showed that the loss of PML in the presence of IFN-β increased the plaquing of ICP0 mutants only 3- to 7-fold compared to control cells (M. Perusina Lanfranca and D. J. Davido, unpublished data). This result was similar to a previous published study showing that PML plays a limited role in IFN-β repression on an ICP0-null mutant (48). Our initial mapping studies indicate that the 427 N-terminal amino acids of ICP0 assist HSV-1 in forming plaques in an established IFN response, where residues from 212 to 427 of ICP0 are necessary for this activity.

### The first 388 amino acids of ICP0 promotes HSV-1 plaquing and viral replication in IFN-β-treated cells.

**FIG 3:**
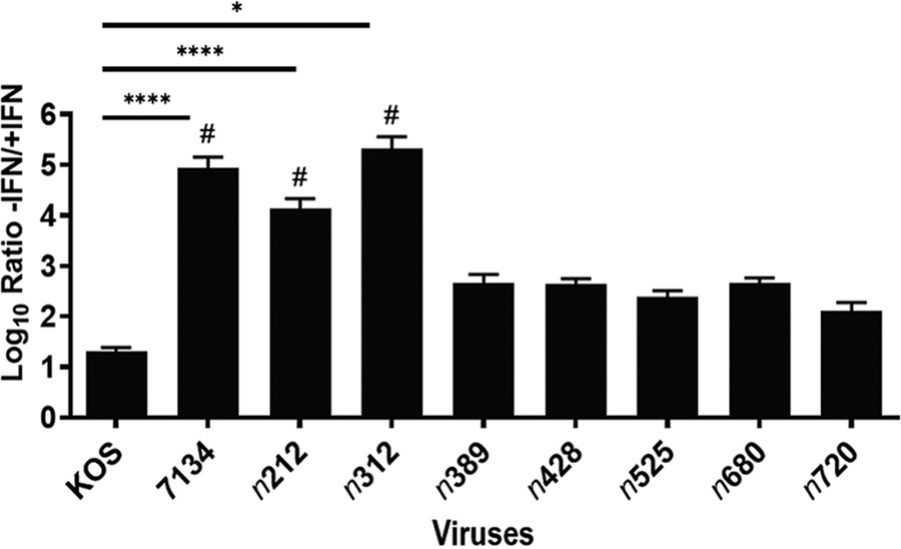
Plaque reduction assays with WT HSV-1 and ICP0 null and ICP0 nonsense linker insertion mutants in HEL cells. HEL cells were infected and treated with or without IFN-β as described in Fig. 2. Calculated ratios correspond to *n *=* *2–4 experiments, with error bars representing SEMs. Pound signs (#) indicate that plaques were not identified for these viruses at the lowest dilution tested with IFN-β and were given the value of 1 for estimating these ratios. *, *P = *0.0473 and ****, *P* < 0.0001 relative to KOS (one-way ANOVA).

To better define the subregion of ICP0 in its N-terminus that allows HSV-1 to form plaques in the presence of IFN-β, we tested two additional truncations mutants generated in our laboratory, *n*312 and *n*389 (30). *n*312 codes for the first 311 amino acids of ICP0, whereas *n*389 codes for its first 388 amino acids (Fig. 1B). These linker insertion viruses were tested with all other viruses examined in Fig. 2. As shown in Fig. 3, the plating efficiencies of 7134 (ICP0 null mutant), *n*212, and *n*312 were impaired by IFN-β (no individual plaques were visible); all other viruses that were examined (*n*389 - *n*720 and KOS) formed plaques and were reduced in their plating efficiencies between 21- and 460-fold when cells were exposed to IFN-β.

**FIG 4:**
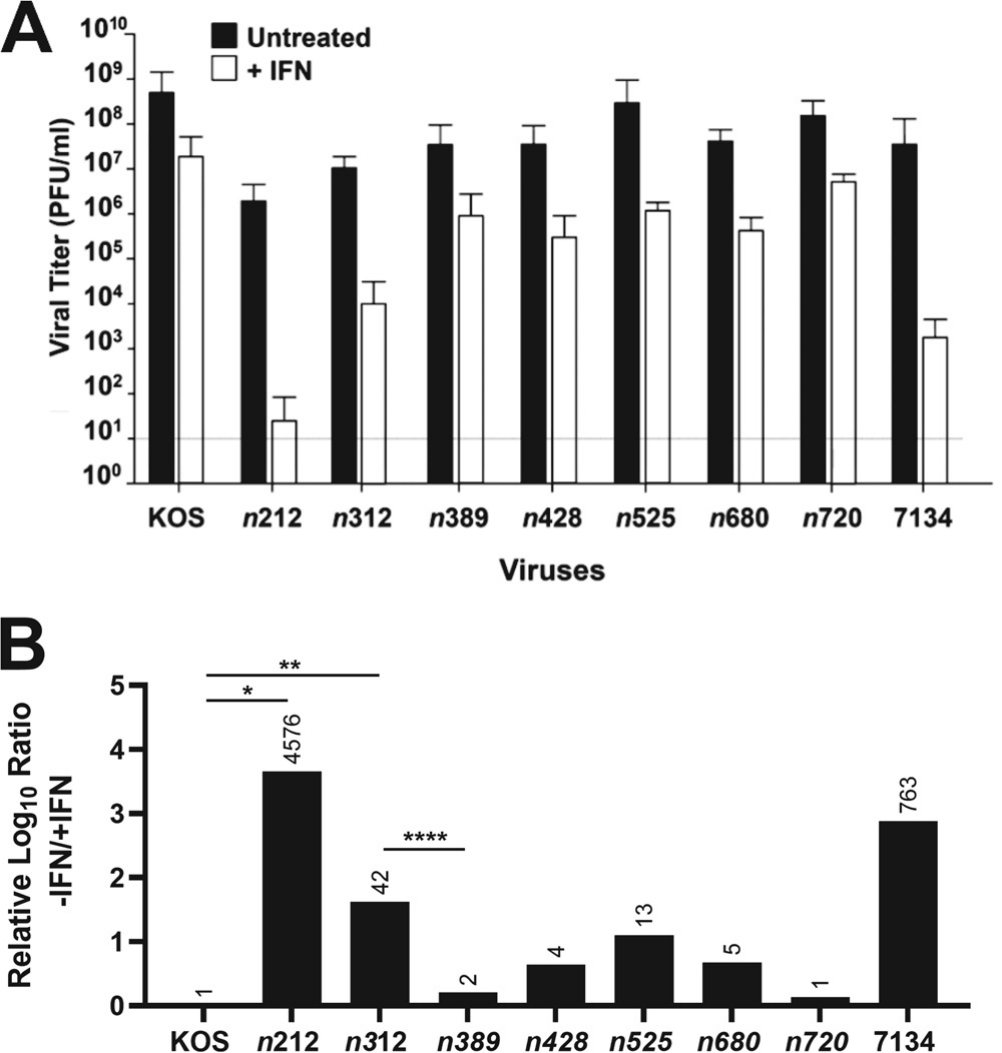
Viral yields of KOS, 7134, and ICP0 C-terminus truncation mutants in HepaRG cells in the presence and absence of IFN-β. (A) HepaRG cells were pretreated or not with IFN-β (1000 U/mL) and were infected 16 hours posttreatment with KOS, 7134, or ICP0 truncation mutants at 1 PFU/cell. Samples were harvested 24 hpi and titered by plaque assays in *n *≥* *4 experiments. Error bars are SEMs. The dashed line is the limit of detection. (B) Ratio of viral yields, −/+IFN-β, for a specific virus based on data from part A compared to KOS, which was given the relative value of 1. *, *P = *0.019, **, *P = *0.0035, and ***, *P = *0.035 (unpaired *t*-test).

To determine if the phenotypes observed in our IFN-β plaque reduction assays correlated with reductions in viral replication, we performed viral growth assays in the absence and presence of IFN-β. Using the same viruses as in the plaque reduction assays (Fig. 3), HEL or HepaRG cells were infected at an MOI of 1. HepaRG cells are human liver epithelial cells, which are easy to culture and responsive to type 1 IFNs and ICP0-null mutants are significantly impaired for viral replication (49–51). We used both cell types in viral assays, but HEL cells were unfortunately too sensitive to the acid washes used to inactivate unabsorbed extracellular virus. Our plaquing phenotypes on HepaRG cells with IFN-β were similar to our results on HEL cells (J. M. van Loben Sels, M. Perusina Lanfranca, and D. J. Davido, unpublished data). Consequently, we only performed our yield assays in HepaRG cells. Results from the viral growth assays (Fig. 4A) correlated well with the plaque reduction assays in Fig. 2 and 3, distinguishing the viral replication phenotypes of *n*312 and *n*389. Comparing the fold differences or ratios in viral replication (-IFN-β/+IFN-β) relative to KOS (WT HSV-1, given value of 1), *n*720 to *n*428 ranged from 1- to 13-fold reductions (Fig. 4B). *n*389 had a 2-fold further decrease in the efficiency of viral replication with respect to KOS, whereas for *n*312, the difference was 42-fold and statistically different from *n*389 (Fig. 4B). We noticed that IFN-β did not restrict *n*312 replication to the same extent as *n*212, whereas IFN-β was able to restrict both viruses well in plaque reduction assays (Fig. 2 and 3). The degree of this restriction is likely due to differences between the time frame (1 versus 3 days) and MOI used in these assays. Overall, these data (Fig. 3 and 4) indicate the first 388 N-terminal amino acids of ICP0 expressed by HSV-1 is sufficient to allow for substantial viral replication when the type 1 IFN response has been stimulated in HepaRG and HEL cells. Furthermore, residues 312 to 388 (a 77 amino acid region) in the N-terminal half of ICP0 appear to attenuate the antiviral effects of IFN-β on HSV-1 replication.

### IFN-β decreases IE, E, and/or L viral gene expression of *n*212 and *n*312 truncation mutants in HepaRG cells.

**FIG 5:**
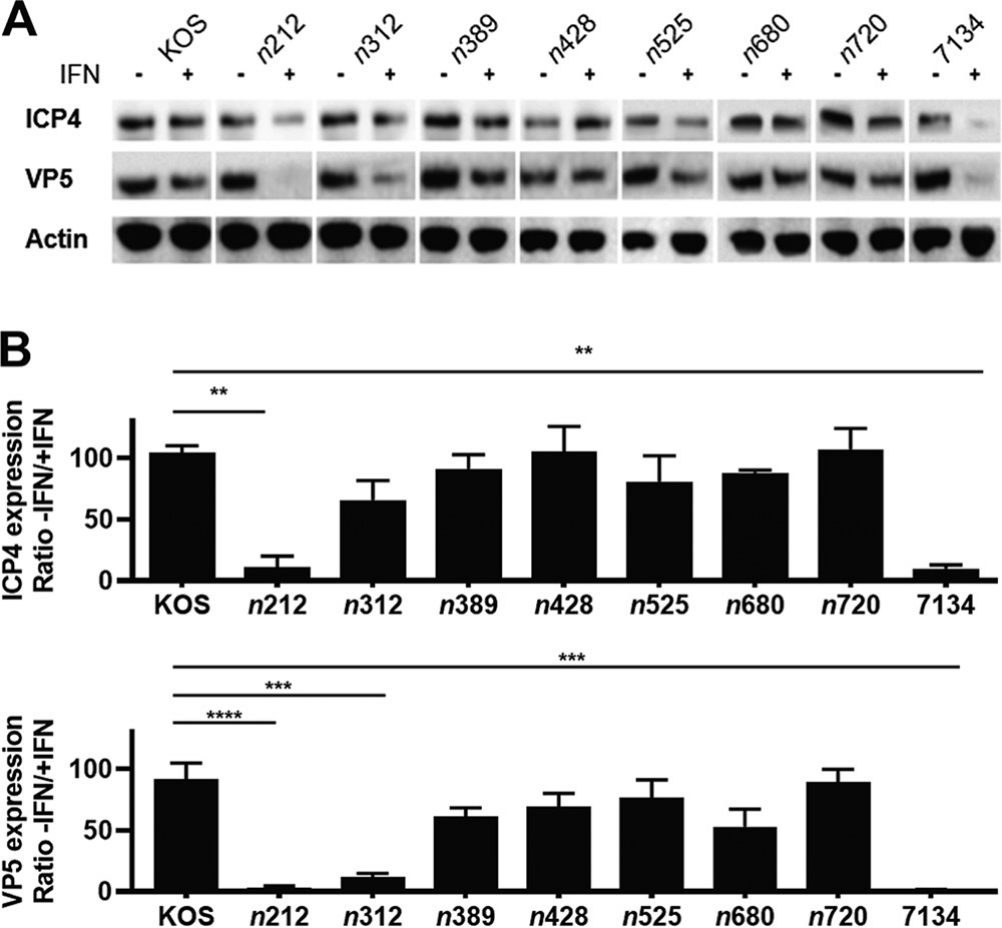
Levels of HSV-1 IE and L proteins in cells infected with ICP0 truncation mutants in cells −/+ IFN-β. HepaRG cells were untreated or pretreated with IFN-β (1000 U/mL) for 16 hours and infected at an MOI of 2.5 with KOS or each ICP0 mutant in the absence (−) or presence (+) of IFN-β. Virally-infected cells were harvested and lysed at 36 hpi, and ICP4 (IE) and VP5 (L) protein levels were determined by Western blots. β-actin levels were included as loading controls. The bar graphs with error bars (SEMs) below the Western blots represent the relative difference (−/+IFN-β) in ICP4 and VP5 protein levels (as measured by densitometry) for each virus tested, which were normalized to β-actin levels. Results were from 3 independent experiments; a representative set of Western blot images is shown above. **, *P = *0.001, ***, *P = *0.0001, and ****, *P* ≤ 0.0001 compared to KOS (one-way ANOVA).

ICP0, as previously described, is a multifunctional protein that acts as a strong transactivator of all kinetic classes of viral genes and with ICP4 synergistically activates E and L genes (41, 52–55). ICP0 contains a large region in its N-terminal half that has been shown to stimulate HSV-1 promoters and gene expression (Fig. 1) (7, 56). Given the overlap between this transactivation domain and our findings in the ICP0-IFN-β replication studies, we wanted to determine if reductions in viral replication correlated with reduced viral protein and transcript levels. To investigate this possibility, HepaRG cells were mock-treated or pretreated with IFN-β for 15 hours and subsequently infected with wild type HSV-1 (KOS) or ICP0 null (7134) or truncation mutants for 36 hours postinfection. Infected cell extracts were examined for representative IE (ICP4) and L (VP5) proteins, using actin as a loading control. As shown in Fig. 5A, ICP4 levels were similar for all viruses in the presence IFN-β compared to KOS, with the exception of *n*212 and 7134 (Fig. 5B). VP5 levels were significantly lower in *n*212-, *n*312-, and 7134-infected cells treated with IFN-β relative to KOS (Fig. 5A and B), whereas all other viruses expressed comparable levels of VP5.

**FIG 6:**
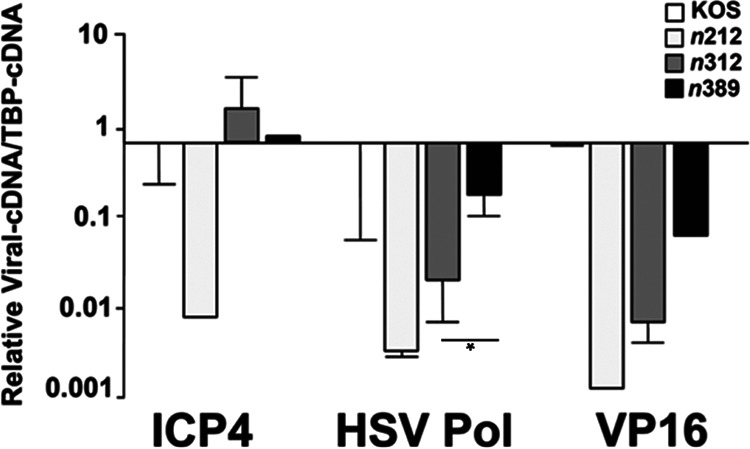
Relative HSV-1 transcript levels in cells treated with IFN-β and infected with WT HSV-1 and ICP0 mutants. HepaRG cells were mock-treated or exposed to IFN-β (1000 U/mL) for 16 hours and infected at an MOI of 2.5 with KOS, *n*212, *n*312, or *n*389 in the presence of IFN-β. Cells were harvested at 24 hpi for RNA, and (IE) ICP4, (E) DNA polymerase, and (L) VP16 RNA levels were quantified by real-time PCR. Viral cDNA levels were normalized to endogenous hTBP; the relative quantities of viral transcripts for KOS were set as the baseline (value 1). Graphs are data compiled from two independent experiments. Error bars represent SEMs. *, *P = *0.032 for DNA polymerase between *n*312 and *n*389 (unpaired *t*-test).

We went on to examine transcript levels of IE (ICP4), E (DNA polymerase), and L (VP16) genes using established and recently developed real-time PCR reagents (57) in cells pretreated with IFN-β and infected with KOS, *n*212, *n*312, or *n*389 for 24 hours (Fig. 6). ICP4, DNA polymerase, and VP16 transcript levels were reduced 75–470 fold in *n*212-infected cells relative to KOS. For *n*312, ICP4 levels were similar to KOS, whereas DNA polymerase and VP16 RNA levels were reduced 25- and 90-fold, respectively. HSV-1 transcript levels were the same (ICP4) or moderately reduced 3- to 9-fold (DNA polymerase and VP16) in *n*389-infected cell + IFN-β compared to KOS. IFN-β noticeably diminished DNA polymerase and VP16 transcript levels a further ~10-fold for *n*312 compared to *n*389, with DNA polymerase being significantly different between these two viruses (Fig. 6). Collectively, the expression levels of E and L genes (Fig. 5 and 6) correlated well with the impairment of *n*212 and *n*312 replication and plaquing by IFN-β.

**FIG 7:**
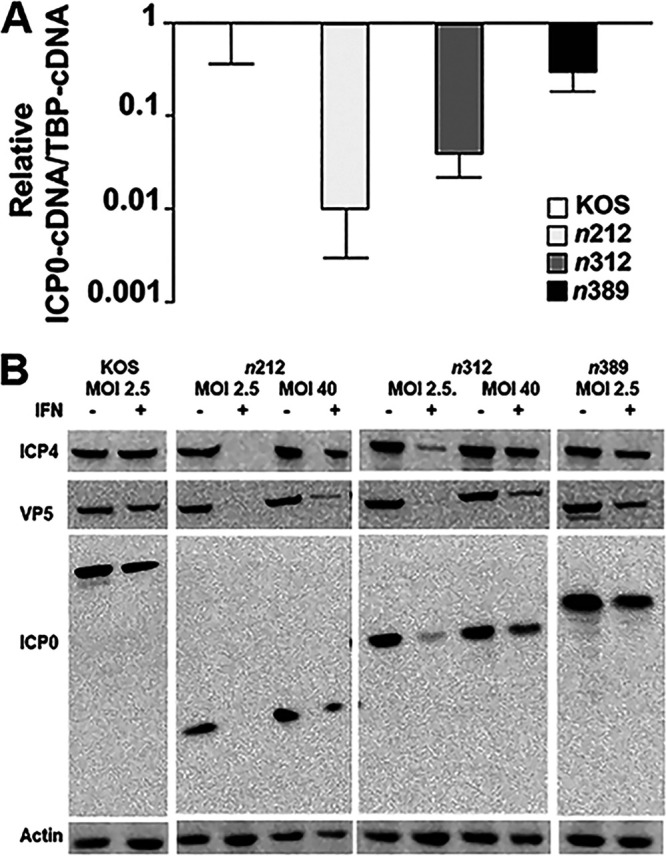
ICP0 and viral gene expression in WT HSV-1 and ICP0 truncation mutants in the presence of IFN-β. (A) HepaRG cells were infected at an MOI of 2.5 with KOS or an ICP0 truncation mutant (*n*212, *n*312, or *n*389) in mock-treated or cells pre-exposed to IFN-β (1000 U/mL). Cells were harvested at 24 hpi to analyze ICP0 RNA levels by quantitative real-time PCR. Viral cDNA levels were normalized to endogenous TBP and compared to transcript levels of ICP0 in KOS. Graphs shown are data compiled from 2 sets of independent experiments. Error bars represent SEMs. (B) HepaRG cells were infected at an MOI of 2.5 with KOS, *n*212, *n*312, or *n*389 or infected at an MOI of 40 with *n*212 or *n*312 in untreated or IFN-β-pretreated cultures. Cells were lysed at 36 hpi to examine ICP0, ICP4, or VP5 protein levels by Western blots. β-actin was included as a loading control. A representative set of images from 3 independent experiments is shown.

We then investigated whether reductions in viral replication directed by IFN-β were linked to diminished ICP0 transcript and protein levels for *n*212 and *n*312 compared to *n*389. As presented in Fig. 7A, we observed that ICP0 transcript levels were highly diminished in *n*212-infected samples (~100-fold) upon IFN-β treatment, noticeably diminished in *n*312-infected samples (25-fold), and only modestly reduced in *n*389-infected cells (3-fold) when compared to WT HSV-1 (KOS). ICP0 protein levels correlated well with transcript levels for these viruses (Fig. 7B). We then examined if an increase in input virus (MOI of 2.5 versus 40) for *n*212 and *n*312 would lead to elevated ICP0 levels capable of stimulating ICP4 and VP5 protein levels. While each mutant form of ICP0 expressed was observed in *n*212- and *n*312-infected cells and resulted in elevated levels of ICP4 at an MOI of 40 in the presence of IFN-β, VP5 expression was limited for both viruses, in contrast to KOS and *n*389-infected cells at an MOI of 2.5 (Fig. 7B). These data strongly suggest that IFN-β mediates the repression of ICP0 transcription, and consequently ICP0 protein expression, during *n*212 infection at a lower MOI. Furthermore, although the mutant form of ICP0 in *n*312-infected cells was more abundant at an MOI of 40 in IFN-β-stimulated cells, its level of expression was not sufficient to appreciably induce VP5 protein levels. This latter observation suggests that this mutant form of ICP0 is unable to overcome the restriction imposed by IFN-β, which was not observed at a lower MOI with the mutant *n*389. We conclude that the 388 residues in the N-terminal half of ICP0 enables HSV-1 to resist an established IFN-β response by stimulating viral gene expression, with residues from 312 to 388 playing an important role in this function.

**FIG 8:**
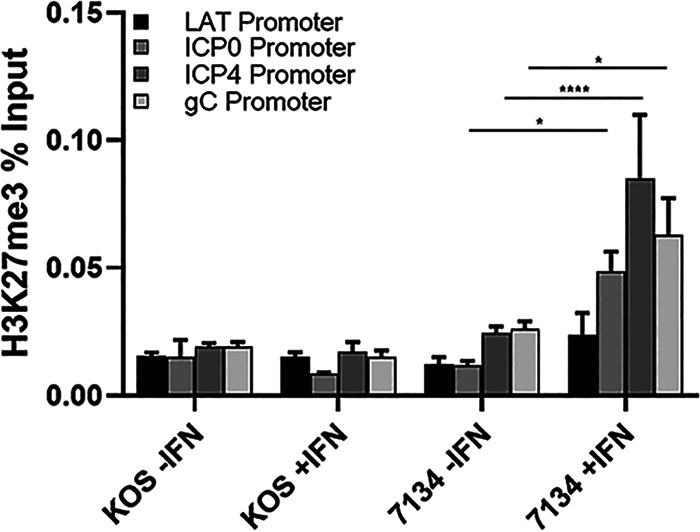
IFN-β increases H3K27 trimethylation on viral lytic promoters of the ICP0-null mutant, 7134. HepaRG cells were untreated or exposed to IFN-β (1000 U/mL) for 15 hours and then infected with KOS (WT HSV-1) or 7134 (ICP0-null mutant) at an MOI of 2.5 for 24 hours. Samples were processed for ChIP assays using a H3K27 trimethylation antibody, quantifying relative H3K27me3 binding to HSV-1 LAT, ICP0, ICP4, and gC promoters by real-time PCR. Mean values for each promoter are shown (*n *=* *4); error bars indicate SEMs. *, *P < *0.013 and ****, *P* < 0.0001 compared to 7134 without IFN-β treatment (two-way ANOVA with Tukey’s multiple comparison test).

Given that ICP0 and chromatin play roles in regulating viral gene expression, we next examined if IFN-β would promote heterochromatin formation on lytic HSV-1 promoters in the absence of ICP0. To test this possibility, we performed chromatin immunoprecipitation (ChIP) analyses in infected cells that were or were not exposed to IFN-β. Our results showed that IFN-β treatment significantly enriched the repressive histone 3 lysine 27 trimethylation (H3K27me3) modification 3- to 6-fold on IE (ICP0 and ICP4) and L (gC) promoters for the ICP0 null mutant (7134) compared to WT HSV-1, strain KOS (Fig. 8). The latency-associated transcript (LAT) promoter was marginally enriched in H3K27me3 markings for the ICP0-null mutant. Thus, IFN-β appears to enhance heterochromatin formation at HSV-1 lytic promoters in the absence of ICP0, linking type 1 IFN response to repressive viral chromatin formation and gene expression.

## DISCUSSION

HSV-1 undergoes lytic and latent infections in its host, establishing a lifelong infection that enables immune evasion and perpetuation. Not surprisingly, HSV-1 has developed counter-defenses which partially or completely inactivate various host antiviral measures. The IE protein ICP0 has been shown to play an important role in evasion of the host immune response. In this study, ICP0 has been shown to assist HSV-1 in overcoming the restriction in viral replication imposed by type 1 interferons (33, 39, 58–61), which are among the first host defense proteins to be synthesized upon viral infection.

The roles of key regions and motifs in ICP0, for example its RING-finger domain (14, 62), nuclear localization signal (63, 64), and ND10 localization domain (62, 65), have been determined relative to ICP0’s E3 ubiquitin ligase activity, transactivation activity, and viral replication. However, identifying domains or regions in ICP0 that are involved in its ability to counteract the type 1 interferon response have been limited. In order to begin to understand how ICP0 facilitates HSV from impairing a preexisting type 1 IFN response, we used a series of viruses expressing progressive C-terminal truncated forms of ICP0. These mutants were used in plaque reduction and viral yield assays in the presence and absence of IFN-β. From such experiments, we observed that the abilities of these mutants to inactivate the IFN response were approximately inversely proportional to their ICP0 amino acid length. Initial mapping studies led us to identify the region between amino acids 212–427 as a key contributor to resisting the antiviral state induced by IFN-β (Fig. 2). Plating and viral yield assays in combination with finer mapping studies (Fig. 3 and 4) lead us to identify the region from amino acids 1–388 of ICP0 (Fig. 3 and 4) as contributing to efficient viral replication in the face of a type I IFN response. It is interesting to note that the lack of the NLS domain of ICP0 did not seem to noticeably affect plating efficiency of some ICP0 mutants (e.g., *n*389 and *n*428), although these mutant forms of ICP0 have been shown to be localized in the nucleus and cytoplasm of infected cell (66).

We did observe that decreases in viral replication for *n*212 and *n*312 by IFN-β directly correlates with reductions in L protein (Fig. 5) and E and/or L transcript levels (Fig. 6) when compared to WT HSV-1 and *n*389, suggesting that these mutant forms of ICP0 are unable to efficiently initiate E and L viral gene expression in the presence of IFN-β. A plausible explanation for the reduction in E and L gene expression with *n*212 are linked to concurrent reductions in ICP0 and ICP4 (IE) transcript levels that we observed, which has been reported for another type 1 IFN (IFN-α) (67). Notably, as ICP4 is the major transactivator of HSV and acts synergistically with ICP0 to stimulate E and L genes, it is consistent that decreases in ICP0 and ICP4 expression observed with *n*212 + IFN-β at an MOI of 2.5 impact E and L gene expression and viral replication. These latter defects by *n*212 could not be overcome using a higher MOI, when ICP4 and ICP0 gene products were expressed, suggesting that a block in viral gene expression occurs after the induction of IE expression.

For *n*312, expression of ICP4 and n312 (ICP0) mutant form was observed in the presence of IFN-β (Fig. 5, 6, and 7), especially at the higher MOI we tested (Fig. 7B). We did observed that n312 protein expression is decreased in the presence of IFN-β at the lower MOI tested (Fig. 7B) and appears to be tied to reductions in ICP0 transcription (Fig. 7A), although its levels may also be affected by protein stability. The possibility that n312 is misfolded seems unlikely, as mutants of ICP0 that contain the first 211 to 241 amino acids still possess ubiquitination activity *in vitro* and/or in cell culture (11, 14). The block in the viral life cycle by IFN-β for *n*312 appears to occur in part at the point of E transcription (Fig. 6), thereby diminishing L gene expression and HSV growth, which is not observed to the same extent with *n*389. Our analyses with ICP0 mutants strongly suggest that amino acids 312–388 in the N-terminal portion of ICP0 play a key role in overcoming the suppression of viral replication by IFN-β. Known functional motifs in this portion of ICP0 include region II of phosphorylation (18) and SIM-like sequence 4 (SLS-4) (see Fig. 1) (15, 16). Recent data indicate that the proximity of region II of ICP0 phosphorylation adjacent to SLS-4 acts as a phospho-SIM (68, 69); phospho-SIMs have been reported to enhance SUMO binding and isoform selectivity (70, 71). An initial protein homology search (e.g., BLAST) of this 77-amino acid region did not identify other highly conserved amino acid sequences from eukaryotic organisms aside from HSV-1 and HSV-2.

A role of SUMOylation in HSV-1 life cycle has been noted as inhibition of SUMO conjugation in the absence of ICP0 that relieves host antiviral resistance to HSV-1 replication (16). This same study showed that the SIMs of WT ICP0 interact with SUMO isoforms and assist in the degradation of SUMO chains or SUMO-modified proteins. A link between SUMO and IFNs is very plausible given that the induction of poly-SUMO chains and conjugation of SUMO isoforms on proteins, including ND10 associated proteins, are induced or elevated in response to type 1 IFNs (72, 73). From our data generated with *n*389, we propose that IFN-β stimulates the expression of poly-SUMO chains and/or SUMO conjugation on cellular restriction factors to repress viral transcription and downstream events in the viral life cycle. As ICP0 is a SUMO-targeted ubiquitin ligase (STUbL), we propose that SLS-4 (as a phospho-SIM) participates in the ubiquitination and subsequent degradation and/or dispersal of SUMO restriction factors to promote E and/or L gene expression. While our unpublished data and one additional study that depleted PML (and its SUMOylated forms) in human cultured cells indicated that PML plays a very limited role in the antiviral repression of type 1 IFNs (52), it is possible that other ISGs that are SUMO-modified may mediate this process. Links between SUMOylation, chromatin, and transcriptional regulation also appear to be plausible, as SUMO isoforms and/or SUMO ligases have been reported to be adjacent to or colocalize with HSV genomes (74–76), as well as a subset of repressed cellular promoter regions (77, 78). Furthermore, we established a link between IFN-β and HSV-1 repressive chromatin (in the absence of ICP0) in this study (Fig. 8); a similar observation has been observed with another DNA virus, hepatitis B virus (HBV), in which IFN-α treatment lead to repressive epigenetic changes on HBV’s cccDNA chromosome and diminished viral transcription (79). Ultimately, understanding how IFNs decrease viral transcript levels and the role of SUMOylation in this process as well as their inactivation by ICP0 will likely provide significant insights into how HSV-host interactions regulate lytic infection, latency, and reactivation.

## MATERIALS AND METHODS

### Cells and viruses.

HEL-299 cells and Vero (African green monkey kidney) cells were maintained at 37°C in 5% CO_2_. HEL-299 cells were cultured in alpha minimum essential medium (αMEM) containing 10% fetal bovine serum supplemented with penicillin (100 U/mL), streptomycin (100 μg/mL), and 2 mM l-glutamine; Vero cells were cultured in Dulbecco’s modified Eagle medium containing 5% fetal bovine serum supplemented with penicillin (100 U/mL), streptomycin (100 μg/mL), and 2 mM l-glutamine. HepaRG (a human hepatocyte-like cell line [36]) were maintained in William’s E media containing 10% FBS, 2 mM l-glutamine, 10 U/mL penicillin, 0.5 μM hydrocortisone, and 5 μg/mL insulin. L7 cells (Vero cells stably transformed with the ICP0 gene) were grown and maintained as previously described (39). WT HSV-1 (strain KOS), 7134 (an ICP0 null mutant), and ICP0 truncation mutants (*n*212, *n*312, *n*389, *n*428, *n*525, *n*680, and *n*720) were propagated as previously described (7, 52). Viral titers for KOS were determined on Vero cells and titers for all ICP0 mutant viruses were determined on L7 cells by standard plaque assays.

### Plaque reduction assays.

HEL-299, HepaRG, Vero, or L7 cells were plated in 24-well plates at 1 × 10^5^ cells/well. The next day, cells were untreated or treated with 1000 U/mL of human IFN-β (AbD Serotec) in their respective medium. After 15 hour of IFN-treatment, cells were infected with 10-fold serial dilutions of the respective WT HSV-1 and ICP0 mutant viruses. After a 1 hour of incubation at 37°C, cells were overlaid with cell culture medium containing 0.5% methylcellulose with or without IFN-β (1000 U/mL). After 3 days postinfection, cultures were washed with PBS and fixed with 3.7% formaldehyde, probed 1 hour at RT (room temperature) with rabbit anti-HSV-1 polyclonal antibody and another hour with a horseradish peroxidase conjugated anti-rabbit (Jackson Immunoresearch). Plaques were visualized with Vector Red substrate (Vector Labs). Stained plates were imaged with a scanner (Cannon), and plaque numbers and image processing were done using Image J (Rasband, W.S., ImageJ, U.S. National Institutes of Health, Bethesda, MD, http://imagej.nih.gov/ij/, 1997–2014) and Adobe Photoshop software. The ratio was calculated as the titer of the viral stock (PFU/mL) in the absence of IFN-β over the titer of each viral stock in the presence of IFN-β. Error bars represent the standard errors of the means.

### Viral Growth Yield Assays.

HepaRG cells were plated in 12-well plates at 1 × 10^5^ cells/well, and the next day cells were either mock-treated or not with 1000 U/mL of human IFN-β (AbD Serotec) 15 hours prior to infection. Cells were infected with HSV-1 or ICP0 mutants at an MOI of 1 for 1h, and acid wash treated to inactivate unabsorbed viruses (80), adding back medium in the absence or presence of 1000 U/mL of human IFN-β. After 24 hours of infection, samples were harvested and frozen at −80°C. The titer of each viral sample was determined on Vero (KOS) or L7 (ICP0 truncation and deletion mutants) cells, respectively.

### RT qPCR.

**ISG transcript analyses.** HEL-299 and HepaRG cells were plated at 1 × 10^5^ cells per well. Next day cells were treated or not treated with human IFN-β at 1000 U/mL. After three PBS washes, cells were harvested in TRIzol (Invitrogen), and cDNA synthesized with iScript cDNA synthesis kit (Bio-Rad). For each sample, real-time PCR was performed using FastStart SYBR green master (Rox) (Roche) in a StepOnePlus real-time PCR system (Applied Biosystems). Transcripts were amplified using the following primer sets: hTBP (5′-TGCACAGGAGCCAAGAGTGAA-3′ and 5′-CACATCACAGCTCCCCACCA-3′), and ISG15 (5′-GGTGGACAAATGCGACGAAC-3′ and 5′-ATGCTGGTGGAGGCCCTTAG-3′) as previous described (81). hTBP RNA levels were used as loading controls between samples (82, 83).

**Viral transcript analyses.** HepaRG cells (5 × 10^5^/well in 6-well plates) were infected with indicated viruses as an MOI of 2.5. At 24 hours postinfection, RNA was purified from HepaRG cells using TRIzol (Invitrogen). To minimize contamination by DNA, we performed a DNase treatment using a TurboDNase kit (Ambion, Life Technologies). cDNA was synthesized using an Omniscript RT kit (Qiagen) and random decamers. Real time PCR was performed using TaqMan Fast Universal qPCR Master Mix (Applied Biosystems) in a StepOnePlus Real Time PCR system (Applied Biosystems). Viral transcripts were amplified using the following primers and probes: ICP4 (fwd: 5′-CACGGGCCGCTTCAC-3′, rev: 5′-GCGATAGCGCGCGTAGA-3′, probe: 5′-CCGACGCGACCTCC-3′), ICP0 cDNA (fwd: 5′-CACCACGGACGAGGATGAC-3′, rev: 5′-GGCGGGCGGTACGT-3′, probe: 5′-ACCTGGACGAAGCAGACT-3′), HSV Pol (fwd: 5′-AGAGGGACATCCAGGACTTTGT-3′, rev: 5′-CAGGCGCTTGTTGGTGTAC-3′, probe: 5′-ACCGCCGAACTGAGCA-3′), and VP16 (fwd: 5′-CTTGGTCGACGAGCTGTTTG-3′, rev: 5′-GCCCCGTTGCGTACAG-3′, probe: 5’CCGTCCGCGTTCATG-3′). All viral quantities were normalized to levels of hTBP using a Gene Expression Assay (Applied Biosystems).

### Western blots.

HEL-299 cells or HepaRG cells were plated at 5 × 10^5^/well in 6-well plates. The next day, cells were mock-treated or treated with 1,000 U/mL of human IFN-β for 15 hours. Following pretreatment cells were infected at an MOI of 2.5 or 40 with KOS or ICP0 mutant virus in the absence or presence of IFN-β (1000 U/mL of human beta interferon). At 36 h postinfection (hpi), the cells were washed 3 times with PBS prior to being scraped into boiling 1X Laemmli buffer containing protease inhibitors (1 μg/mL aprotinin, 1 μg/mL leupeptin, 1 mM phenylmethylsulfonyl fluoride), vortexed, and heated for additional 5 min at 95°C. 7.5% of each sample was resolved on a 4–12% Bis-Tris gradient gel (Invitrogen) and subsequently transferred to nitrocellulose membranes. Membranes were blocked for 1 hour at room temperature with 5% BSA in Tris-buffered saline with 0.1% Tween 20 (TBS-T). Blots were probed for protein detection with primary antibody (diluted 1:1000 in blocking buffer) overnight at 4°C (ICP0: H11060, Santa Cruz Biotechnology; ICP5 or VP5: 56989; ICP4: H1A021, EastCoast; Actin: I-19, Santa Cruz Biotechnology) in blocking buffer. After three washes with TBS-T, membranes were probed with either goat-anti-mouse or goat anti-rabbit IgG-horseradish peroxidase (HRP) (Jackson ImmunoResearch). Blots were washed an additional three times with TBS-T and were developed using ECL reagents (SuperSignal West Femto or Pico Chemiluminescent Substrate, Thermo Fisher Scientific). When applicable, probed membranes were stripped in 100 mM 2-mercaptoethanol, 62.5 mM, (Tris pH 6.7), 2% SDS for 21 min at 50°C. Blots were again blocked and probed overnight with antibodies diluted in blocking buffer that recognize actin (I-19, Santa Cruz Biotechnology). After washing, the membrane was incubated with goat-anti-rabbit IgG-HRP (Jackson ImmunoResearch) and developed as previously described. All images were assembled using Adobe Photoshop and Adobe Illustrator software.

### ChIP assays.

ChIP assays to examine histone H3 lysine 27 trimethylation (H3K27me3) was carried out in a similar manner as previously described (84). Briefly samples were kept at 4°C and treated with HALT protease inhibitor (Thermo Fisher) until de-cross-linking. Cells were cross-linked using formaldehyde (final 1% concentration) and stopped by the addition of glycine [0.125M]. Cell were washed and lysed in sodium dodecyl sulfate (SDS) solution. Fixed cells were sonicated to shear DNA. 10% of each sample was retained as an input control, with the remaining part of each sample incubated overnight with an H3K27me3 antibody (10 μg/μL, Millipore 07–449) at 4°C. H3K27me3 antibody-containing complexes were isolated with protein A/G-magnetic beads (Millipore) and then washed. Protein-DNA complexes were eluted in a 0.1% SDS and 1 M NaHCO_3_ solution at 65°C and cross-links were removed in a 0.2M NaCl solution at 55°C for 4 h. Samples were incubated with RNase A and proteinase K, and DNA was isolated with a QIAquick PCR purification kit (Qiagen). Real time PCR was performed using TaqMan Fast Universal qPCR Master Mix (Applied Biosystems) in a StepOnePlus Real Time PCR system (Applied Biosystems). Viral DNA was amplified using the following primers specific for the ICP0, ICP4, LAT, and gC promoters (84).
